# Analysis of endophyte diversity of *Gentiana officinalis* among different tissue types and ages and their association with four medicinal secondary metabolites

**DOI:** 10.7717/peerj.13949

**Published:** 2022-08-30

**Authors:** QinZheng Hou, DaWei Chen, Yu-pei Wang, Nurbiye Ehmet, Jing Ma, Kun Sun

**Affiliations:** 1 The Northwest Normal University, Lanzhou, China; 2 Gansu Provincial Maternity and Child-Care Hospital, Lanzhou, China

**Keywords:** *G. officinalis*, Endophytes, Diversity, Secondary metabolite, Correlation analysis, Ecological function

## Abstract

**Background:**

The difference of metabolites in medicinal plants has always been concerned to be influenced by external environmental factors. However, the relationship between endophytes and host metabolites remains unclear.

**Methods:**

In this study, we used 16S and ITS amplicon sequencing to compare endophyte diversity among different tissue types and ages of *Gentiana officinalis*. Endophyte diversity and abundance was also analyzed in relation to the abundance of four secondary metabolites (Gentiopicroside, Loganic acid, Swertiamarine and Sweroside).

**Results:**

The diversity and richness of *G. officinalis* endophyte differed as a function of tissue types and ages. Four metabolites of *G. officinalis* were significantly correlated with the abundance of dominant endophyte genera. The predictive function analysis showed that metabolism was main function of endophytic bacteria in different tissue and year root samples, while saprotroph was dominant trophic modes of endophytic fungi in the different year root samples. The dominant trophic modes of endophytic fungi was saprotroph and pathotroph, and relative abundances differed in the different tissue samples. The results of this study will help to elucidate the plant-microbial interactions and provide key information on the role of endophytes in the production of *G.officinalis* and its important metabolites.

## Introduction

Endophytes are non-pathogenic microorganisms that live inside a plant (Arnold et al., 2003). Although the roles of most endophytes are unknown, some endophytes are known to play a vital role in plant growth, development, and stress tolerance (Márquez et al., 2007). Previous work also demonstrates that endophytes may impact host secondary metabolites production (Wani et al., 2016; Compant et al., 2016). For example, Chen et al. (2021) reported that the five secondary metabolites of *R. palmatum* were positively correlated with the diversity and abundance of endophytic fungi and Song et al. (2017) reported that endophytic *Bacillus altitudinis* can improve ginsenoside accumulation of *Panax ginseng*. Gao et al. (2015) reported that endophytic *Paenibacillus polymyxa* improved plant growth, increased ginsenoside content, and reduced morbidity of *P. ginseng*.

*Gentiana officinalis* H. Smith (also called Qinjiao in Chinese; Cao & Wang, 2010), has been used for medical purposes for 2,000 years, and was frequently used as a composition in some traditional formulae (Tao et al., 2018). The main active ingredient of the Qinjiao include gentiopicroside, loganic acid, swertiamarine and sweroside, which are used as standards to measure the quality of Qinjiao (Cao & Wang, 2010). Qinjiao has many biological and pharmacological effects, such as stomachic, choleretic and antihepatotoxic activities (Yang et al., 2020). Furthermore, it has anti-inflammatory, antifungal and antihistamine activities, which is recorded in the National Pharmacopoeia of China (Yin et al., 2009). In the past few years, the wild resources of Qinjiao are declining faster than ever, with most natural populations being destroyed to meet the commercial demand (Cao et al., 2005). Therefore, it was important to better understand Qinjiao biology and identify scientific practices to replace or supplement the traditional modes of Qinjiao cultivation.

Endophytes often play a key role in medicinal plants (Abdulazeez, Jeffrey & Johannes, 2020). However, few studies have explored the diversity and function of the endophytes of *G. officinalis*. Therefore, the objectives of this study were as follows: (1) compare the diversity of endophytes among different tissue types and ages of *G. officinalis*; (2) predict the functions of endophyte in *G. officinalis*; and (3) determine whether there is a relationship between endophyte abundance and the abundance of host metabolites. These results may expand the knowledge of plant-microbe relationships and the production of secondary metabolites key to the quality of *G. officinalis*.

## Materials and Methods

### Experimental materials

To compare the diversity of endophytes in *G. officinalis*, three tissue types (*i.e*., leaf, stem and root) were collected from 3-year old plants and in order to compare the effect of plant age on endophytes diversity, root samples were collected from 1, 3 and 5 year old *G. officinalis* plants grown in Tianzhu county, Wuwei city, Gansu Province, China (102°33′34″E, 34°58′1″N). Three biological replicates were collected for each age category. The different tissue samples were divided and washed with water and then rinsed 3× with distilled water. Washed tissues were successively submerged in 75% ethanol for 5 min, followed by 2.5% sodium hypochlorite (NaClO) solution for 2 min, and 75% ethanol for 1 min, and rinsed five times with sterile water. The final sterile water was inoculated in potato dextrose agar medium (PDA) and nutrient agar medium (NA) and the plates were incubated at 28 °C for 10 d and 37 °C for 5 d respectively to evaluate surface-disinfection effect. All disinfected samples were stored at −80 °C until processed.

### DNA extraction, PCR (polymerase chain reaction) amplification, and sequencing

The total genomic DNA of all samples was extracted by using the MOBIO Power-Soil^®^ Kit (MOBIO Laboratories, Inc., Carlsbad, CA, USA), according to the manufacturer’s instructions. The DNA concentration was estimated by NanoDrop spectrophotometer (Model 2000; Thermo Fisher Scientific, Waltham, MA, USA) and stored at −20 °C for PCR. The 20 μL mixture of PCR assays included 4 μL of 5× Fast-Pfu buffer, 2 μL of 2.5 mM dNTPs, 0.8 μL of each primer (5 μM), 0.4 μL of FastPfu Polymerase, ca. 10 ng of templateDNA and ddH_2_O. The bacterial 16S rDNA gene (V3–V4 region) was amplified with primers (Castrillo et al., 2017): 338F (5′-ACTCCTACGGGAGGCAGCA-3′) and 806R (5′-GGACTACHVGGGTWTCTAAT-3′), according to the following thermocycler conditions: 3 min of initial denaturation at 95 °C, 30 cycles of 30 s at 95 °C, 52°C for 30 s, and 72 °C for 45 s, and final extension of 5 min at 72 °C. The fungal ITS1 rDNA region was amplified using the ITS primers: ITS1F (5′-CTTGGTCATTTAGAGGAAGTAA-3′) (Gardes & Bruns, 1993) and ITS2R (5′-GCTGCGTTCTTCATCGATGC-3′) (Jiao et al., 2022). ITS PCR reaction were performed with the following thermocyler program: denaturation at 95 °C for 3 min, 35 cycles at 95 °C for 30 s, 30 s for annealing at 55 °C, and 45 s for elongation at 72 °C, and final extension of 10 min at 72 °C. The PCR products were visualized with 2% agarose gel electrophoresis. Successful PCR products of all sample were pooled and purified using EasyPureTM PCR Cleanup/Gel Extraction Kit (Axygen Biosciences, Union City, CA, USA) according to manufacturer’s instructions. All samples were amplified in triplicate and pooled prior to sequencing. Purified PCR products were sequenced on an Illumina NovaSeq platform (Caporaso et al., 2012).

### Metabolites of *G. officinalis* quantitative analysis

Standards of gentiopicroside, loganic acid, swertiamarine and sweroside were purchased from Shanghai R&D Center for Standardization of Traditional Chinese Medicines. High-performance liquid chromatography (HPLC)-ultrapure water, analytical-grade methanol and phosphoric acid were purchased from Sangon Biotech, Ltd. (Shanghai, China).

The dried plant samples (*i.e*., subset of the same tissue that was surface sterilized and used for DNA extraction) were pulverized and sieved through a 300 μm mesh. A total of 1.0 g of powdered samples were weighed and 20 mL methanol was added and treated with ultrasound (30–40 °C, 250 W, 50 kHZ) for 30 min. Filtrate was obtained by filtration of 0.22 μm Millipore filter unit, and 10 μL of sample solution was injected into HPLC for determination. Samples were analyzed by HPLC (Waters) using C18 (4.6 × 250 mm, 5.0 μm, Waters E2695; Milford, MA, USA) at 30 °C, and the content of metabolites were determined: The mobile phase was methanol (A) −0.15% phosphoric acid (B). 0–4 min, 25% A; 4–12 min, 25–33% A; 12–20 min, 33–40% A; 20–25 min, 40–25% A. The flow rate was 1 mL·min^−1^. The detection wavelength was 242 nm.

### Data analysis

According to methods of Penton et al. (2016), QIIME (V1.9.1, http://qiime.org/scripts/split_libraries_fastq.html) was used to analyze the data (Caporaso et al., 2010). Fungal and bacterial sequences were first trimmed and assigned to each sample based on unique barcodes sequences. Sequences were binned into operational taxonomic units (OTUs) at a 97% similarity level with UPARSE software (UPARSE v7.0.1001, http://drive5.com/uparse/) (Tanja & Steven, 2011). The bacterial OTUs were classified at the species level by searching for all sequences that match the Silva bacterial 16S database (Tanja & Steven, 2011), and fungal OTUs were classified at the species level by searching in the UNITE database (Mejía et al., 2008) after removing low confidence classifications. Rarefaction based on Mothur v.1.21.1 was used to analyze the diversity indices, including goods_coverage, Chao 1 and Shannon (Schloss et al., 2009). Community differences among samples was analyzed by using UPGMA (Unweighted pair group method with arithmetic mean) cluster analysis (*e.g*., Aliyu et al., 2018). Predicted functions of each OTU were estimated with PICRUSt for the bacterial 16S rDNA (ref to PICRUSt) and FUNGuild v1.0 (ref to FUNGuild) for the fungal ITS (*e.g*., Chen et al., 2020). Spearman method was used to analyze the correlation between metabolite abundance and endophyte abundance (Stevens, Wellner & Acevedo, 2010). All experiments were carried out with least three independent replicates. All of the data were expressed as mean ± standard error. All data were analyzed by one-way analysis of variance (ANOVA) and the differences among the means were compared by Duncan’s multiple range test with a significance of *p* < 0.05 using SPSS 16.0 statistical program.

## Results

### Surface-sterilization efficiency

After cultivation, we found that no colonies appeared in PDA and NA medium, which illustrates that surface-sterilization of samples was effective and this methodology may be used for subsequent experiments.

### Analysis of sequencing data and alpha diversity

A total of 186,871 and 194,319 and 199,441 and 179,183 effective tags were obtained for 16S and ITS sequencing of the different types of tissue and the different age root, respectively. The goods_coverage of the all samples were higher than 0.977, which indicates that the sequencing data can fully reflect the community structure of endophytes (Table 1).

**Table 1:** Community diversity of endophytic fungi and bacteria of different tissue and year samples.

Sample	Endophytic fungi					Endophytic bacteria			
	Effective tags	Shannon	Chao1	Goods_coverage		Effective tags	Shannon	Chao1	Goods_coverage
Root	65,831	5.324	622.993	0.998		63,353	4.464	422.295	0.977
Stem	57,866	6.201	811.540	0.998		66,169	2.322	203.481	0.990
Leaf	63,174	6.029	724.601	0.998		64,797	2.588	149.525	0.991
1^st^ year root	65,726	3.879	508.313	0.998		53,285	6.242	1461.193	0.984
3^rd^ year root	65,831	5.458	635.207	0.998		63,353	4.487	467.111	0.998
5^th^ year root	67,884	4.644	596.456	0.998		62,545	4.755	621.643	0.995

**Figure 1:**
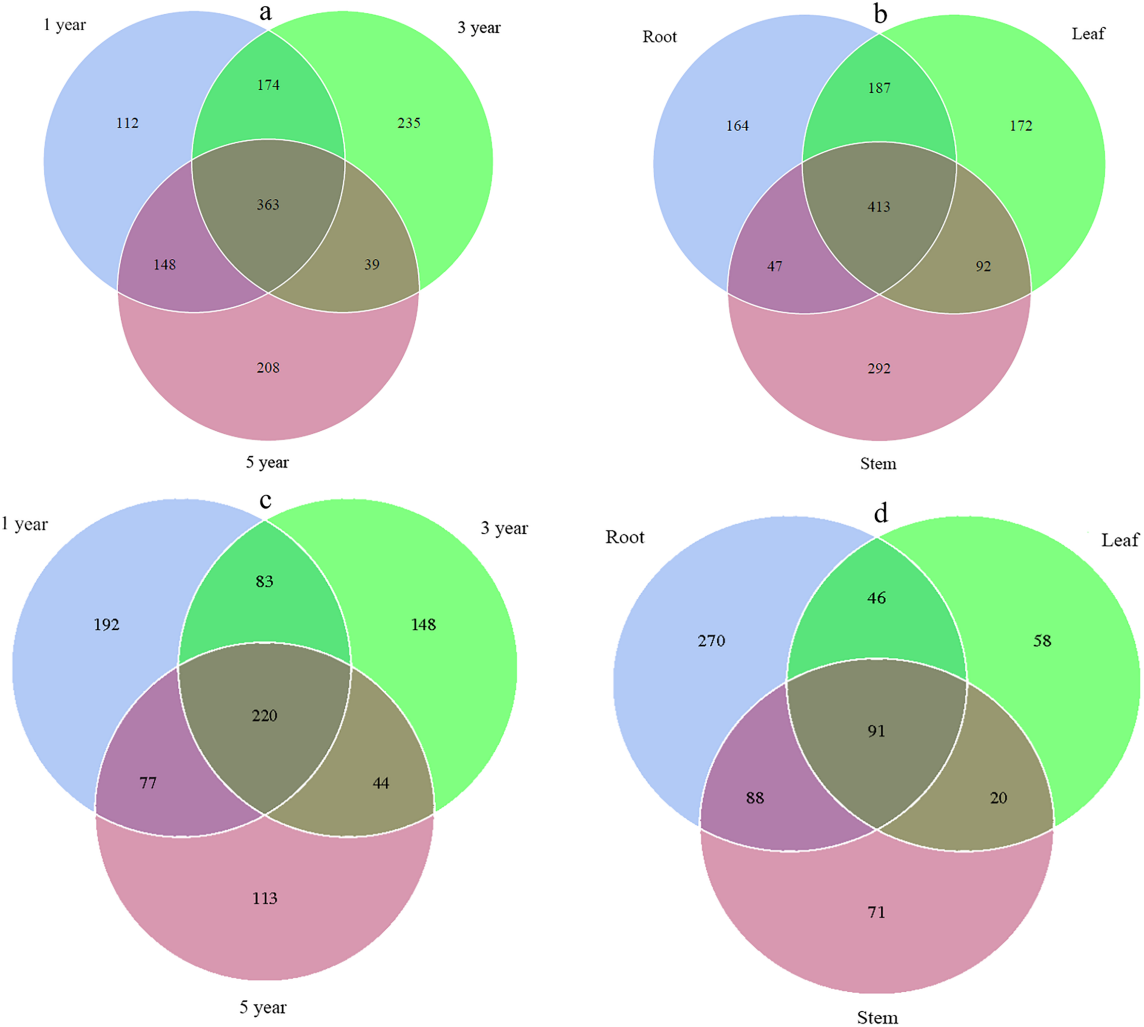
Venn diagram showing the fungal OTUs of different year samples (A), fungal OTUs of different tissue samples (B), bacterial OTUs of different year samples (C) and bacterial OTUs of different tissue samples (D).

Across all libraries, 363 fungal ITS OTUs and 220 bacterial 16S OTUs were shared among root samples collected in different years. The numbers of fungal ITS OTUs that occurred uniquely in the first, third and fifth year root samples were 112, 235, and 208, respectively, while the numbers of bacterial 16S OTUs were 192, 148, and 113, respectively (Figs. 1A and 1C). Overall, 413 fungal ITS OTUs and 91 bacterial 16S OTUs were shared among different tissue samples. The numbers of fungal ITS OTUs that occurred uniquely in root, stem, and leaf samples were 164, 292, and 172, respectively, while the numbers of bacterial 16S OTUs that occurred only in root, stem, and leaf samples were 270, 58, and 71, respectively (Figs. 1B and 1D).

Alpha diversity indices (Chao1 and Shannon’s diversity index) differed among all samples of *G. officinalis*. In the different tissue samples, the fungal communities’ richness and diversity was highest in stem, followed by leaf and root, while the bacterial communities’ diversity of root was highest, followed by leaf and stem, the bacterial communities’ richness of root, followed by stem and leaf (Table 1). In the different year root samples, the fungal communities’ richness and diversity was highest in the third year root, followed by fifth and first year root samples. The bacterial communities’ richness and diversity was highest in the first year root, followed by the fifth and third year root samples (Table 1).

### Composition of fungal and bacterial communities

**Figure 2:**
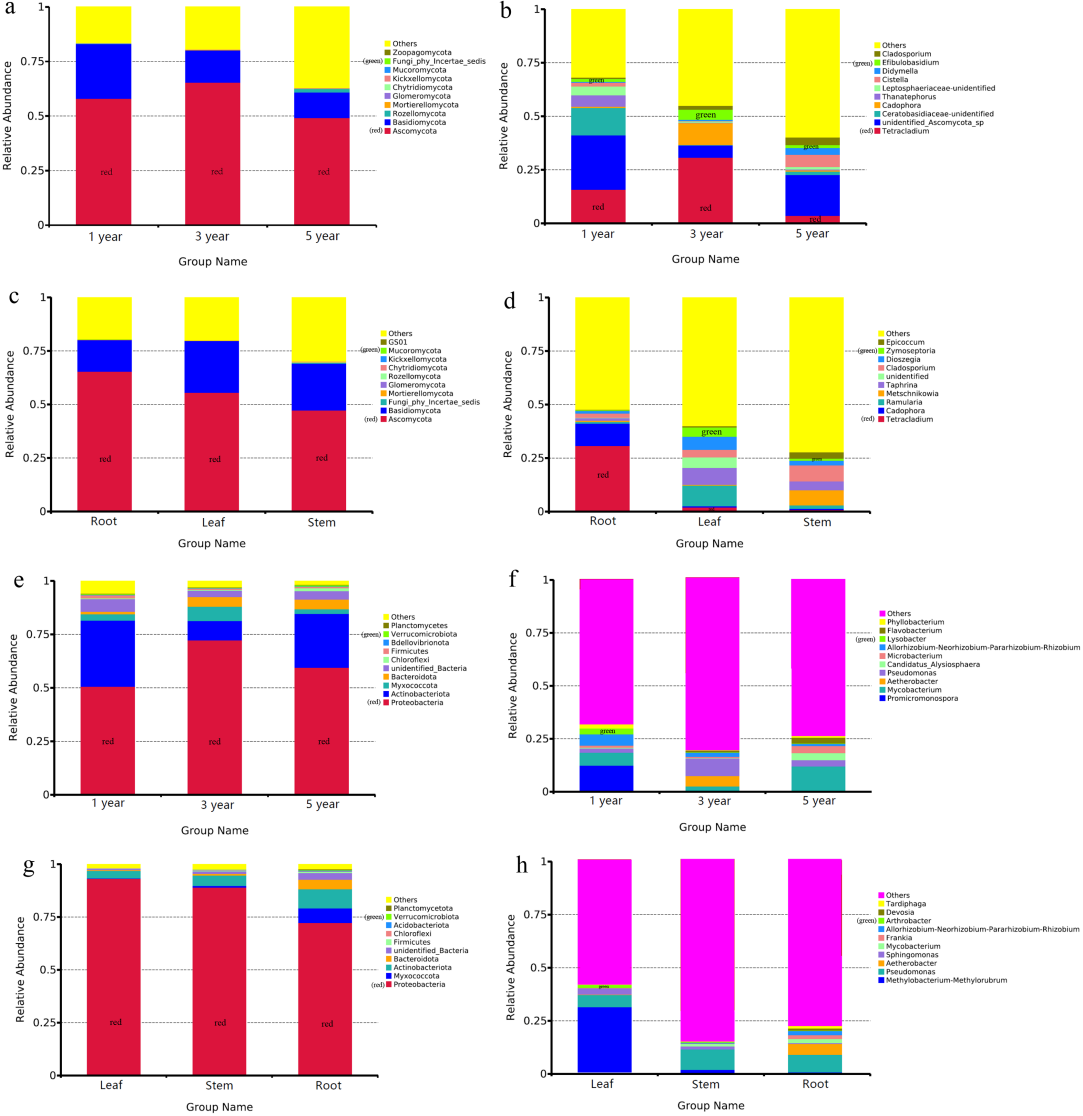
Relative abundances of the endophyte. Note: fungal phylum of different year root samples (A), fungal genus of different year root samples (B), fungal phylum of different tissue samples (C), fungal genus of different tissue samples (D), bacterial phylum of different year root samples (E), bacterial genus of different year root samples (F), bacterial phylum of different tissue samples (G) and bacterial genus of different tissue samples (H). Relative abundances are based on the proportional frequencies of the DNA sequences that could be classified. “Other” represents the total of relative abundance outside top ten maximum relative abundance levels.

The OTUs of endophytic fungi were assigned into 14 phyla and 327 genera in different year root samples. The relative community abundance of the top ten fungal phyla at the phylum level is shown in Fig. 2A. Ascomycota was dominant fungal phylum in the first, third and fifth year root sample, with relative abundances of 49.20% to 65.41%. At the genus level, *unidentified_Ascomycota_sp* was dominant genus in the first and fifth year root samples (25.37% and 19.09%), *Tetracladium* was dominant genus inthird year root samples (30.87%) (Fig. 2B). In the different tissue samples, the fungal OTUs were assigned into 13 phyla and 342 genera. Ascomycota was dominant fungal phylum in the root, stem and leaf sample, with relative abundances ranging from 47.40% to 65.40% (Fig. 2C). At the genus level, *Tetracladium* was dominant genus in root samples (30.87%), *Ramularia* was dominant genus in leaf samples (9.62%) and *Cladosporium* was dominant genus in root samples (7.56%) (Fig. 2D).

Bacterial OTUs were assigned into 40 phyla and 314 genera in different year root samples. The dominant bacterial phylum across different year root samples were Proteobacteria, with relative abundances ranging from 50.76% to 72.32% (Fig. 2E). At the genus level, *Promicromonospora* was dominant genus in the first year root samples (12.15%), Pseudomonas was dominant genus in the third year root samples (8.28%) and *Mycobacterium* was dominant genus in the fifth year root samples (11.73%) (Fig. 2F). In the different tissue samples, the bacterial OTUs were assigned into 27 phyla and 251 genera. Proteobacteria was dominant bacterial phylum in the different tissue samples, with relative abundances ranging from 72.41% to 93.22% (Fig. 2G). At the genus level, *Methylobacterium-Methylorubrum* was dominant genus in leaf samples (30.76%), *Pseudomonas* was dominant genus in stem and root samples (9.82% and 8.24%) (Fig. 2H).

**Figure 3:**
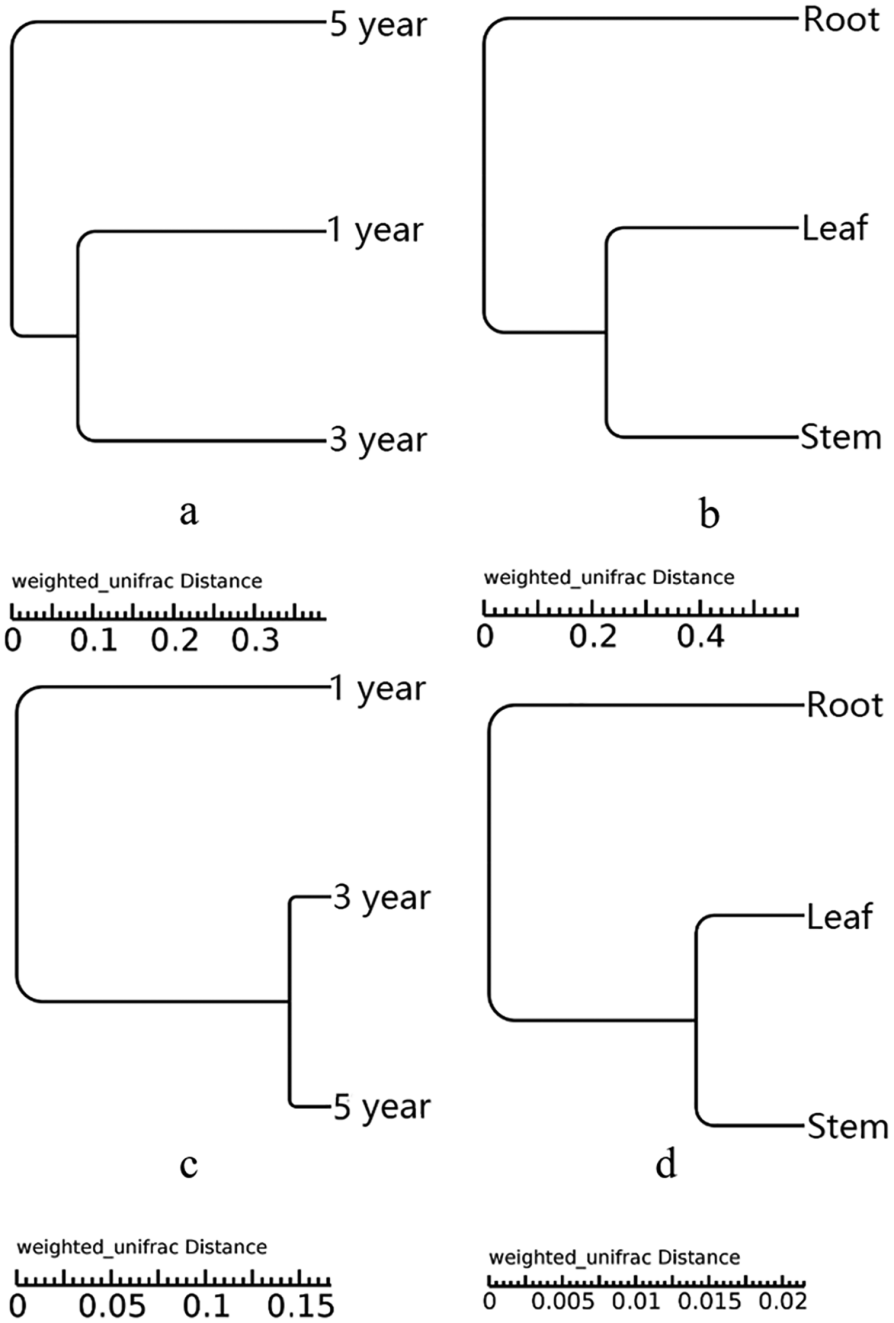
UPGMA tree of fungi of different year root samples (A), fungi of different tissue samples (B), bacteria of different year root samples (C) and bacteria of different tissue samples (D).

UPGMA showed that all the samples were grouped into two different clusters (Fig. 3). The root samples were clustered into group 1, and the samples of leaf and stem were clustered into group 2. The UPGMA tree result indicated that the fungal and bacterial compositions of stem and leaf samples were more similar than the root samples (Figs. 3B and 3D). The fifth year root samples were clustered into group 1, the first and third year root samples were clustered into group 2 in the fungal compositions (Fig. 3A). While the first year root samples were clustered into group 1, the third and fifth year root samples were clustered into group 2 in the bacterial compositions (Fig. 3C).

### Correlation analysis between endophytes and metabolites of *G. officinalis*

**Figure 4:**
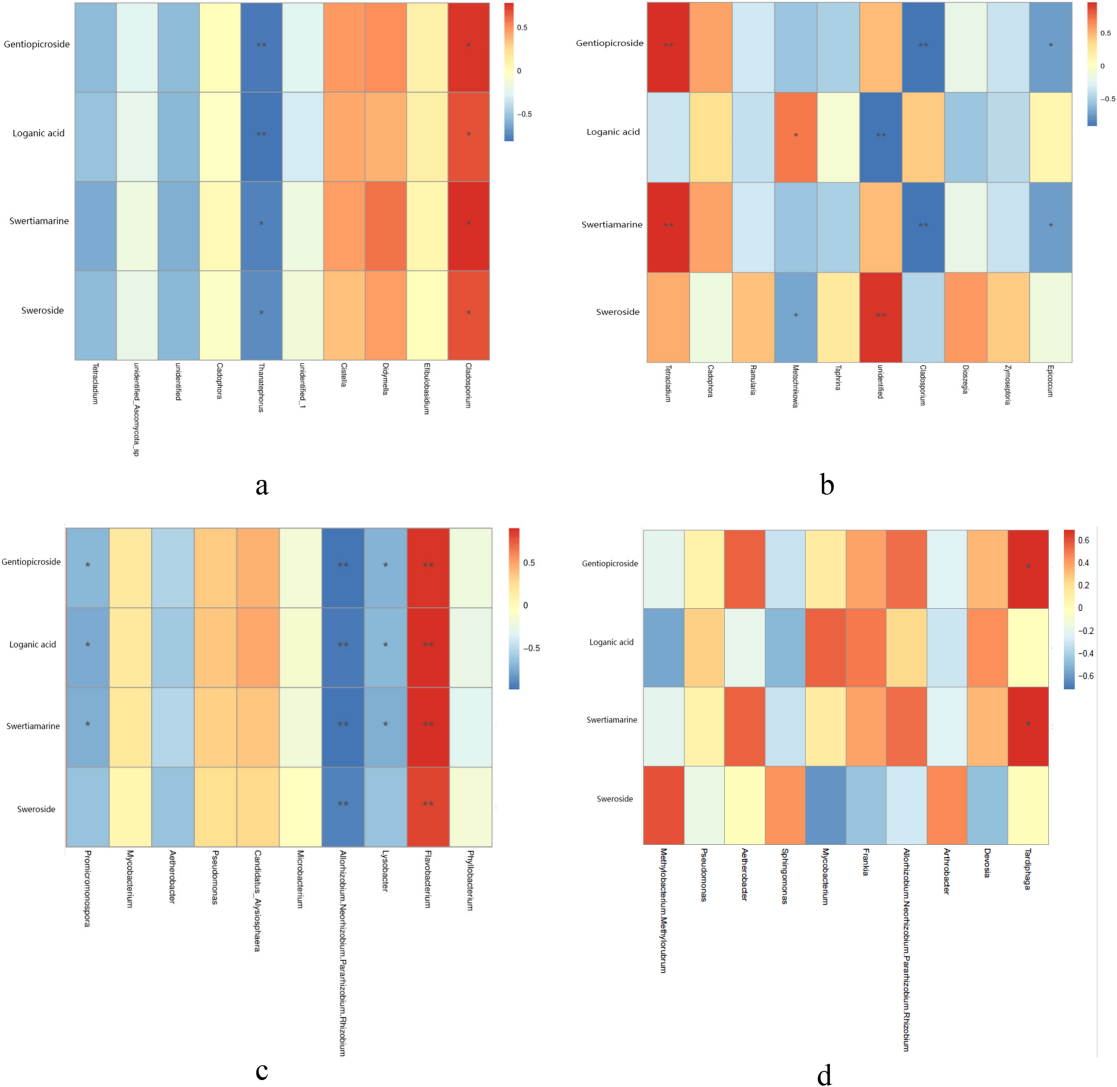
Correlation analysis between metabolites and top 10 maximum relative abundance of endophytes at the genus level. (A) Fungal genus of different year root samples, (B) fungal genus of different tissue samples, (C) bacterial genus of different year root samples, (D) bacterial genus of different tissue samples. An asterisk (*) indicates the differences are significant at *p* < 0.05; two asterisks (**) indicates the differences are significant at *p* < 0.01.

Metabolites content were different in different year root and tissue samples (Table 2). Correlation analysis between metabolites content and endophytic fungi abundance showed that the abundance of four metabolites was significantly positively correlated with *Cladosporium*, while *Thanatephorus* was significantly negatively correlated with four metabolites in the different year root samples (Fig. 4A). In the different tissue samples, *Tetracladium* was significantly positively correlated with the content of gentiopicroside and swertiamarine, *Metschnikowia* was significantly positively correlated with the content of loganic acid, while *Cladosporium* and *Epicoccum* was significantly negatively correlated with the content of gentiopicroside and swertiamarine (Fig. 4B).

**Table 2:** Metabolite content of different year and tissue of *G. officinalis*.

Sample	Gentiopicroside (mg/g)	Loganic acid (mg/g)	Swertiamarine (mg/g)	Sweroside (mg/g)
Root	129.92 ± 1.27 b	7.39 ± 0.09 c	2.52 ± 0.03 b	0.90 ± 0.02 c
Stem	30.88 ± 0.58 e	8.88 ± 0.13 a	0.67 ± 0.04 e	0.29 ± 0.02 e
Leaf	42.59 ± 1.67 d	3.98 ± 0.12 d	0.98 ± 0.04 d	1.08 ± 0.09 b
1^st^ year root	75.67 ± 0.75 c	4.12 ± 0.08 d	1.32 ± 0.01 c	0.48 ± 0.05 d
3^rd^ year root	129.92 ± 1.27 b	7.39 ± 0.09 c	2.52 ± 0.03 b	0.90 ± 0.02 c
5^th^ year root	145.04 ± 1.92 a	7.66 ± 0.12 b	3.03 ± 0.08 a	2.34 ± 0.11 a

**Note:**

Values are mean ± SD (*n* = 3). Different letters indicate the differences are significant at *p* < 0.05.

Correlation analysis of metabolites content and endophytic bacterial abundance showed that the contents of four metabolites in root samples collected from different years was significantly positively correlated with *Flavobacterium*, while *Allorhizobium, Neorhizobium, Pararhizobium, Rhizobium* were significantly negatively correlated with four metabolites. *Lysobacter* and *Promicromonospora* were significantly negatively correlated with the content of gentiopicroside, loganic acid and swertiamarine in the different year root smples (Fig. 4C). In the different tissue samples, *Tardiphaga* was significantly positively correlated with the content of gentiopicroside and swertiamarine (Fig. 4D).

### PICRUST and FUNGuild functional prediction analysis

**Figure 5:**
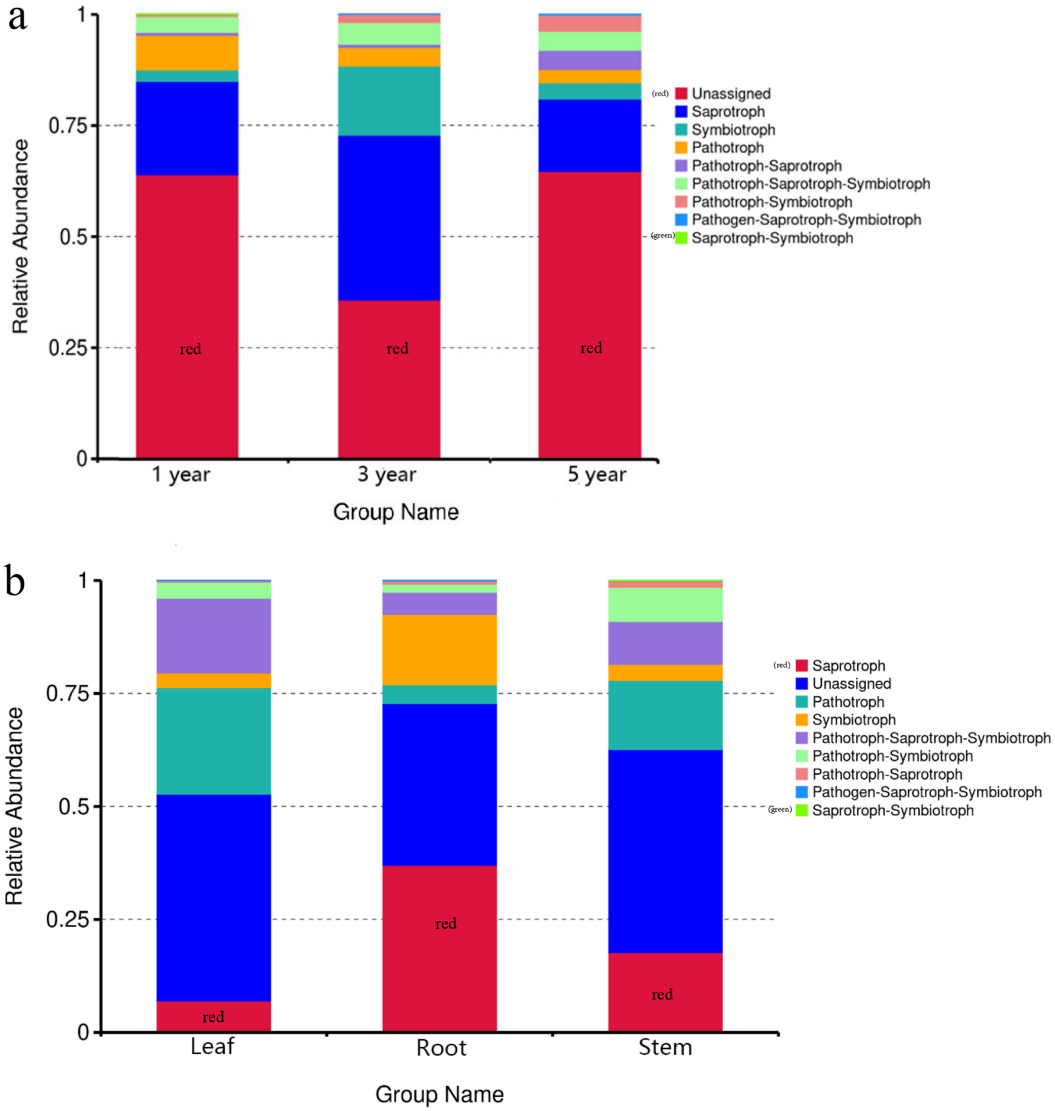
Relative abundance of predicted function of fungi in different year root samples (A), fungi in different tissue samples (B).

**Figure 6:**
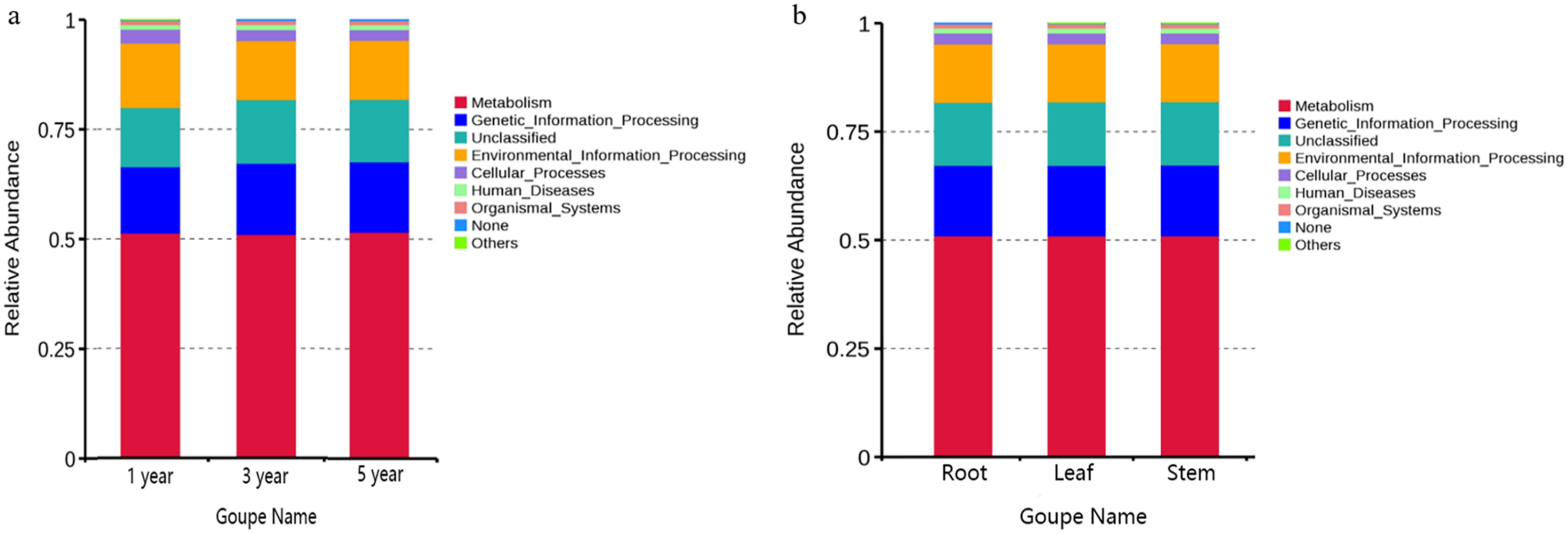
Relative abundance of predicted function of bacteria in different year root samples (A) and bacteria of fungi in different tissue samples (B).

FUNGuild was commonly used to predict the nutritional and functional groups of fungal communities.The results showed that saprotroph was dominant trophic modes in the different year root samples, with relative abundances ranging from 16.27% to 20.96% (Fig. 5A). The trophic mode of endophytic fungi differed in different tissue samples (Fig. 5A). Saprotroph was dominant trophic modes in the root and stem samples (17.73% and 37.02%), while pathotroph was dominant trophic modes in the leaf samples (23.63%) (Fig. 5B). To study bacterial function, we used PICRUSt to perform bacterial function prediction analysis. Through comparison with the Kyoto Encyclopedia of Genes and Genomes (KEGG) database, the PICRUSt analysis of bacterial 16S rDNA sequences showed that metabolism was main function in all samples, accounting for 50.99–51.62% (Figs. 6A and 6B).

## Discussion

### The effect of tissue type and age on diversity

Many studies reported that the diversity of the endophytes are affected by host species, tissue types, as well as plant growth stage (*e.g*., Hassan, 2017). For example, Araújo et al. (2020) reported that fungal diversity of *Hevea brasiliensis* was higher in the stems and roots than in leaves, whereas the fungal abundance was higher in the leaves. However, we found that fungal endophyte diversity and abundance of *G. officinalis* was highest in stem, followed by leaves and roots. We speculated that the plant species influenced the selection of fungal endophytes (Dong et al., 2018). As for endophytic bacteria, we found that bacterial communities’ diversity was highest in root, followed by leaf and stem, and the bacterial communities’ richness was highest in root, followed by stem and leaf. These results were similar with Huo et al. (2020), who found that the diversity and richness of bacterial endophyte of *Glehnia littoralis* root were highest, followed by leaf and stem. Many studies have reported that most bacterial endophyte come from soil (Hardoim et al., 2011). The diversity of bacterial endophyte in roots is higher than that in leaves or stems due to interactions between plants and soil (Hardoim et al., 2011). The results indicated that the fungal and bacterial endophyte among different tissues of medicinal plants was different. In addition, Hong et al. (2019) reported that the diversity of endophytic bacteria in the second year ginseng root tissues was the greatest. While the fungal communities’ richness and diversity of *G. officinalis* was highest in the third year root, followed by the fifth year and the first year root samples. The communities’ richness and diversity of bacteria was highest in the first year root, followed by the fifth year and the third year root samples.

### The effect of tissue type and age on composition

Many studies have reported that the bacterial and fungal communities of plant through HTS method analysis indicated only a few dominant phyla, including bacteria (Proteobacteria, bacteroidetes and actinobacteria) and fungi (Ascomycota, basidiomycota and zygomycota) (Miguel et al., 2016; Gazis & Chaverri, 2010). In this study, the endophytic bacterial and fungal communities of *G. officinalis* different tissue samples were clustered into 27 and 13 phyla respectively, and the dominant phyla of bacteria and fungi were proteobacteria and ascomycota, which is consistent with previous research. Proteobacteria and ascomycota were dominant phylum among the different tissues, but the relative richness differed, which was consistent with the reports of bacterial communities in the *P. notoginseng* and *H. brasiliensis* (Araújo et al., 2020; Dong et al., 2018). However, the dominant fungal and bacterial genera differed significantly in the different tissue samples. This result account for communities of bacteria and fungi having certain tissue specificity. Jin et al. (2014) reported that endophytic bacteria in leaf and stem of *Stellera chamaejasme* grouped together, but root endophytic bacteria differed, our result is consistent with these prior results. While Araújo et al. (2020) reported that the fungal communities of *H. brasiliensis* in roots and stems clustered together, but leaves differed, which is different with our results. The results proved that the endophyte communities can be influenced by different tissues. Furthermore, the endophytic bacterial and fungal communities of different year *G. officinalis* root were clustered into 40 and 14 phyla respectively, and the dominant phyla of bacteria and fungi were proteobacteria and ascomycota, which is same with the previous studies. The results of phylum level analysis showed that dominant phylum of different year samples were proteobacteria and ascomycota, respectively, while the relative abundance differed. This results were similar to the reports of bacterial communities in *P. ginseng* (Hong et al., 2019). However, the dominant fungal and bacterial genera differed significantly in the different year root samples. The results may prove that the endophyte communities can be influenced by plant age.

### Endophyte associations with metabolites

Endophyte have biosynthesis ability, which can produce many bioactive secondary metabolites. Numerous studies have reported that endophytes can produce substitutes same or similar to the secondary metabolites of the host (Zhou et al., 2010; Zhao et al., 2011; Ludwig-Müller, 2015). In this study, spearman method was used to analyze the relationship between endophytes and host secondary metabolites. The results showed that the secondary metabolites of *G. officinalis* were significantly correlated with multiple endophytic fungi and bacteria. This results indicated that plant secondary metabolite synthesis is associated with many endophytes, not just one. However, Chen et al. (2021) and Cui et al. (2018) reported that metabolites content of *Rheum palmatum* and *Cynomorium songaricum* were only correlated with endophytic fungi. The reason for this phenomenon may be related with plant species. Interestingly, the contents of four metabolites was significantly positively correlated with *Cladosporium* in the different year root smples, while *Cladosporium* was significantly negatively correlated with the content of gentiopicroside and swertiamarine in the different tissue samples, this phenomenon may be due to genus existed tissue-specificity.

### Predicted ecological function of endophyte

PICRUSt analysis can predict reliability of the function of bacteria (Langille et al., 2013), and has been used to study the function of endophytic bacteria (Luo et al., 2017). We used results of high-throughput sequencing (HTS) for PICRUSt function prediction analysis. The results showed that the metabolism was main function in all samples. This result is similar to the results of Pii et al. (2016) study on the rhizosphere bacterial function of barley and tomato. Pepe-Ranney et al. (2019) reported that endophyte originated from the rhizosphere microbiome, so it leads to the similar results. These results indicated that the age and tissue of *G. officinalis* did not affect the function of endophytic bacteria.

FUNGuild was used to estimate the fungal ecological functions. Furthermore, it has been used to study the fungal community (Martínez-Diz et al., 2019). The results of FUNGuild suggested that the trophic modes of fungal endophyte in different tissue samples differed, but the trophic modes of fungal endophyte in different year root samples was the same, which indicated that plant ages may not exert an effect on fungal endophytic function of *G. officinalis*. Although FUNGuild has been used to analyze the fungal trophic mode, duo to existing literature and data, this method has some limitations. Therefore, to comprehensively study the function of endophytic fungi, it is necessary to further explore the fungal classification and function in the soil.

## Conclusions

In this study, we found that the diversity and richness of endophyte of *G. officinalis* differed among different tissues and ages, and the four metabolites of *G. officinalis* were significantly correlated with the multiple dominant genus of endophyte. The metabolism was main function of endophytic bacteria in different tissue and year root samples. While saprotroph was dominant trophic modes of endophytic fungi in the different year root samples, the dominant trophic modes of endophytic fungi was saprotroph and pathotroph. The results of this study will help to elucidate the plant-microbial interactions and provide key information on the role of endophytes in the production of *G.officinalis* and its important metabolites.

## Additional Information and Declarations

### DNA Deposition

The following information was supplied regarding the deposition of DNA sequences: The 16S rRNA and ITS gene sequences of endophytes are available at NCBI: SAMN21356694.

### Data Availability

The following information was supplied regarding data availability: The 16S rRNA and ITS gene sequences of endophytes are available at NCBI: SAMN21356694. The metabolite data are available at figshare: Chen, DaWei (2022): Endophyte and secondary metabolites of *Gentiana officinalis*. figshare. Dataset. https://doi.org/10.6084/m9.figshare.20000927.v2.
